# An ensemble learning approach for diabetes prediction using boosting techniques

**DOI:** 10.3389/fgene.2023.1252159

**Published:** 2023-10-26

**Authors:** Shahid Mohammad Ganie, Pijush Kanti Dutta Pramanik, Majid Bashir Malik, Saurav Mallik, Hong Qin

**Affiliations:** ^1^ AI Research Centre, School of Business, Woxsen University, Hyderabad, India; ^2^ School of Computer Applications and Technology, Galgotias University, Greater Noida, India; ^3^ Department of Computer Science, Baba Ghulam Shah Badshah University, Rajauri, India; ^4^ Department of Environmental Health, School of Public Health, Harvard University, Boston, MA, United States; ^5^ College of Engineering and Computer Science, University of Tennessee at Chattanooga, Chattanooga, TN, United States

**Keywords:** diabetes prediction, ensemble learning, XGBoost, CatBoost, LightGBM, AdaBoost, gradient boost

## Abstract

**Introduction:** Diabetes is considered one of the leading healthcare concerns affecting millions worldwide. Taking appropriate action at the earliest stages of the disease depends on early diabetes prediction and identification. To support healthcare providers for better diagnosis and prognosis of diseases, machine learning has been explored in the healthcare industry in recent years.

**Methods:** To predict diabetes, this research has conducted experiments on five boosting algorithms on the Pima diabetes dataset. The dataset was obtained from the University of California, Irvine (UCI) machine learning repository, which contains several important clinical features. Exploratory data analysis was used to identify the characteristics of the dataset. Moreover, upsampling, normalisation, feature selection, and hyperparameter tuning were employed for predictive analytics.

**Results:** The results were analysed using various statistical/machine learning metrics and k-fold cross-validation techniques. Gradient boosting achieved the greatest accuracy rate of 92.85% among all the classifiers. Precision, recall, f1-score, and receiver operating characteristic (ROC) curves were used to further validate the model.

**Discussion:** The suggested model outperformed the current studies in terms of prediction accuracy, demonstrating its applicability to other diseases with similar predicate indications.

## 1 Introduction

Diabetes mellitus is a severe and chronic disease characterised by metabolic disorders in which the pancreas either fails to produce insulin, or the body cannot effectively utilise the insulin produced (Sneha and Gangil, 2019). Lack of awareness about the symptoms and complications of diabetes is prevalent due to limited healthcare resources in many parts of the world (Webber, 2013). There are approximately 40 different types of diabetes, with some common types being Type 1 (insulin-dependent), Type 2 (insulin-independent), gestational diabetes, and pre-diabetes (Kharroubi and Darwish, 2015).

According to statistical reports from various healthcare organisations, it is estimated that globally, 463 million adults, which accounts for 9.3% of the population aged between 20 and 79 years, are affected by this chronic disease (Diabetes Federation International and IDF, 2019). This highlights the widespread prevalence and significance of diabetes as a global health issue.

Projections suggest that the prevalence of diabetes will continue to increase significantly, with an estimated 578 million individuals affected by 2030. According to the Diabetes Atlas 2019 by the International Diabetes Federation (IDF), approximately 50% or 231 million people living with diabetes remain undiagnosed and unaware of their condition due to limited healthcare resources (Diabetes Federation International and IDF, 2019).

In 2019 alone, diabetes was responsible for 4.2 million deaths worldwide. This chronic disease can have detrimental effects on various organs in the human body, including the brain, nerves, heart, kidneys, eyes, and skin. Recognising the symptoms and signs of diabetes is crucial for early detection and management. Some common early symptoms observed in individuals with diabetes or those at risk include excessive thirst, fatigue, unexplained weight gain, dizziness, skin discoloration, sexual dysfunction, fungal infections, high blood sugar levels, and frequent urination (Sneha and Gangil, 2019). These symptoms serve as important indicators for seeking medical attention and further evaluation.

Indeed, given the significant impact and global burden of diabetes, there is an urgent need to leverage computational intelligence techniques for improved prediction and prevention of this disease. By utilising advanced machine learning and artificial intelligence algorithms, we can develop models that can effectively identify individuals at risk of developing diabetes. These models can analyse large-scale datasets, extract meaningful patterns, and generate accurate predictions.

The application of computational intelligence techniques in diabetes prediction can have several benefits. Firstly, it can enable early disease detection, allowing for timely intervention and management strategies. This early detection can aid in preventing or delaying diabetes-related problems, improving overall health outcomes for people.

Furthermore, by accurately predicting diabetes, healthcare professionals can implement preventive measures and provide personalised care plans for high-risk individuals. This can involve lifestyle modifications, dietary interventions, exercise regimens, and medication management to effectively manage and control blood sugar levels.

Overall, applying computational intelligence techniques to diabetes prediction can significantly enhance medical results, lessen the condition’s toll, and encourage proactive and preventative healthcare practices for those at risk.

However, healthcare data are growing drastically, and the traditional machine learning approaches have been found inadequate to handle such voluminous data for accurate disease predictions. Ensemble learning techniques offer better performance in this regard.

This work aims to create a model that accurately predicts diabetes using ensemble learning approaches. Our work’s contribution is as follows:

• Performing exploratory data analysis to improve the dataset’s quality assessment.
• Performing data augmentation and processing using upsampling and data normalisation, respectively.
• Using a k-fold cross-validation procedure to confirm the results.
• Building the model by employing boosting algorithms in conjunction with an ensemble learning strategy.
• Increasing prediction accuracy through hyperparameter tuning.
• Determining the contribution of the features towards diabetes.
• Comparing the proposed model’s performance assessment to other research studies of a similar nature.

The rest of the paper is organised as follows. Related work is discussed in Section 2. The adopted methodology and the dataset are presented in Section 3. Then, the experimental details and results are described and analysed in Section 4. Next, the comparative analysis with existing similar works is presented in Section 5. Lastly, the conclusion and the future direction of the research are provided in Section 6.

## 2 Related work

In recent years, copious work has been done on the prediction of diabetes using machine learning and ensemble learning tools and techniques (Ganie et al., 2022a; Ganie and Malik, 2022a). Different datasets, algorithms, and methodologies used by the researchers to carry out this research work have been discussed. The developed models have yielded better results and can be used to support healthcare providers in data-driven decision-making. This section reviews some of the key relevant papers on applying ensemble learning approaches to forecast diabetes.

Li et al. (2020) developed a model to predict diabetes using ensemble learning techniques to enhance disease prediction using the Pima diabetes dataset. They achieved the highest results with extreme gradient boosting (XGBoost), with an accuracy rate of 80.20%. The authors proposed the improved feature combination classifier using the XGBoost model, which can be explored to better predict diseases in the healthcare industry. Mahabub (2019) tested different ensemble learning techniques, such as AdaBoost, gradient boost, XGBoost, random forest, etc., to predict diabetes, considering several clinical parameters such as pregnancy, skin thickness, glucose, insulin, blood pressure, diabetes pedigree function, body mass index (BMI), age, and class variable (outcome). They achieved the highest accuracy rate of 84.42% with the multilayer perceptron algorithm. Mushtaq et al. (2022) proposed an optimised model using a voting classification based on the ensemble method to predict diabetes using the Pima diabetes dataset. This research work used a two-stage model selection process to develop the model. The voting classifier reached the best accuracy rate of 81.50% among all the classifiers. Furthermore, Tomek and synthetic minority oversampling technique (SMOTE) techniques were used for data balancing to remove the biases from the dataset. The authors suggested that the research be continued to estimate the likelihood that nondiabetic patients will develop this condition in the future.

Beschi Raja et al. (2019) employed different boosting algorithms for the diabetes prediction model development. The gradient boosting algorithm attained the highest accuracy rate of 89.70% among all the classifiers. Other statistical measurements have also been evaluated to validate the proposed model. Khan et al. (2021) developed a model for diabetes prediction using boosting method. The authors explored different classifiers such as gradient boosting, hybrid k-nearest neighbour (kNN), j48, deep learning, naive Bayes, and artificial neural network (ANN) for predictive analytics. Among all the classifiers, the gradient boosting algorithm attained the best results. In addition, the results were validated using the k-fold cross-validation method. The authors suggested that this model can be used as a prognosis tool in the healthcare industry for early disease prediction. Lai et al. (2019) developed a complete framework for the predictive analysis of diabetes. The gradient boosting machine techniques were used with hyperparameter tuning, particularly for class balancing, which minimised the loss of prediction probabilities regarding classification.

Singh et al. (2021) introduced an ensemble approach based framework called eDiaPredict to forecast the diabetes status of patients. The proposed methodology incorporates XGBoost, random forest, support vector machine (SVM), neural network, and decision tree. The efficacy of eDiaPredict is demonstrated through its implementation on the PIMA Indian diabetes dataset, resulting in an attained accuracy, precision, and sensitivity of 95%, 88%, and 90.32%, respectively, with the combination of XGBoost and random forest. Hasan et al. (2020) presented a framework for predicting diabetes using kNN, decision trees, random forest, AdaBoost, Naive Bayes, XGBoost, and multilayer perceptron. They employed a weighted ensemble of the machine learning models to improve the prediction accuracy, and experimented on the PIMA Indian diabetes dataset. The proposed ensemble model achieved a significantly higher AUC and specificity of 0.950 and 0.934, respectively. However, it exhibited lower accuracy, precision and sensitivity of 88.84%, 84.32%, and 78%, respectively.

## 3 Research methodology

**FIGURE 1:**
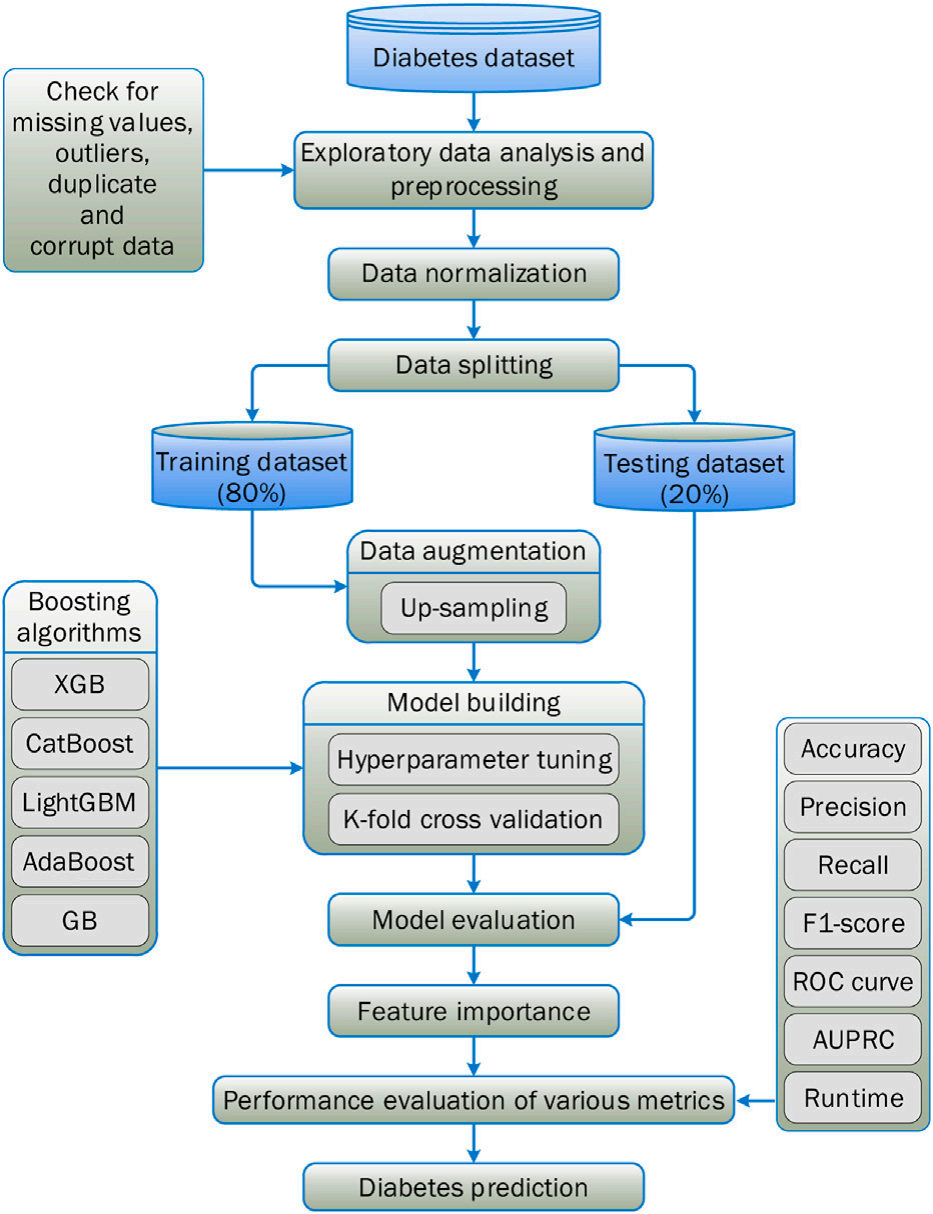
Proposed methodology for research work.

Figure 1 illustrates the procedural flow of the proposed framework employed in this experimental study. It outlines the sequential steps undertaken to enhance the prediction accuracy of diabetes using an ensemble learning technique based on boosting methods. The Pima Indians diabetes dataset, obtained from the Kaggle community, was utilised for this study. Initially, the necessary Python library packages were installed in Jupyter Notebook. Exploratory data analysis was conducted to enhance the dataset’s quality assessment. During this phase, missing values were identified and replaced through data imputation. The Interquartile Range method was applied to detect outliers in the dataset (Ganie et al., 2023).

Other necessary libraries were run to check the dataset for any corrupted data. Upsampling and normalising were also carried out before the five boosting methods under consideration were created. The dataset was split with a ratio of 80:20, where 80% of the data was employed for training the boosting algorithms, and 20% was used to test and validate their efficacy. Hyperparameter tuning was applied during the model-building process for better results.

### 3.1 Boosting algorithms adopted

Ensemble learning has been utilised in several real-life problems (Ganie et al., 2023). In healthcare, ensemble learning has gained significant popularity due to its effectiveness in predicting, detecting, diagnosing, and prognosing various diseases. In this particular experiment focusing on diabetes prediction, we examined the following five boosting algorithms based on ensemble learning:

• **XGBoost:** XGBoost operates by integrating diverse types of decision trees, also known as weak learners, to independently compute similarity scores (Santhanam et al., 2016). By incorporating gradient descent and regularisation techniques, XGBoost effectively addresses the issue of overfitting that can arise during the training phase. Modifying the gradient descent and regularisation procedure, it aids in overcoming the issue of overfitting during the training phase.
• **CatBoost:** The CatBoost short form of categorical boosting is faster than other boosting algorithms, as it does not require the exploration of data preprocessing (Hancock and Khoshgoftaar, 2020). It is used to deal with high cardinality categorical variables. In the case of low cardinality variables, one-hot encoding technique is used for conversion.
• **LightGBM:** Light gradient boosting machine (LightGBM) is an extension of a gradient boosting algorithm capable of handling large datasets with less memory utilisation during the model evaluation process (Machado et al., 2019). Gradient-based one-sided sampling method is used for splitting the data samples, reducing the number of features in sparse datasets during training.
• **AdaBoost:** AdaBoost, also known as adaptive boosting, operates by dynamically adjusting weak learners’ weights without prior knowledge (Sevinc, 2022). During the training process, the weakness of each base learner is evaluated based on the estimator’s error rate. The AdaBoost algorithm commonly employs decision tree stumps to address classification and regression problems.
• **Gradient boosting:** The gradient boosting (GB) method trains weak learners in a sequential manner, with each estimator being added one by one by adjusting their weights (Aziz et al., 2020). This algorithm’s main goal is to forecast residual errors from earlier estimators and reduce the difference between anticipated and actual values. This iterative process allows for continuous improvement in the overall predictive performance.

### 3.2 Attribute information

The dataset consists of 768 instances and nine attributes. The first eight attributes are independent variables, also known as predicates, while the last attribute is the dependent or target variable. Table 1 provides detailed information about the attributes, including their descriptions, measurements, and range values.

**TABLE 1:** Attributes information of the dataset.

Attribute	Description	Measurement	Value range
Pregnancy (PR)	Participant number of times pregnant	Numeric	0–17
Glucose (GL)	Plasma glucose concentration of the participant	mg/dL	0–199
Blood pressure (BP)	Diastolic blood pressure of the participant	mmHg	0–122
Skin thickness (ST)	Triceps skin fold thickness of the participant	mm	0–99
Insulin (IN)	Participant’s insulin level (2-h serum)	(mu U/mL)	0–846
Body mass index (BMI)	Body fat based on the height and weight of the participant	kg/m^2^	0–67
Diabetes pedigree function (DPF)	Likelihood of diabetes based on the family history of the participant	*p*-value	0.07–2.42
Age (AG)	Age of the participant	Years	21–81
Diabetes (DB)	Class attribute	0 = no diabetes, 1 = diabetes	0 or 1

### 3.3 Dataset description

Descriptive statistics are crucial in revealing the characteristics of data samples, summarising information to facilitate human interpretation. Table 2 presents attribute information along with their corresponding measures, including the record count, minimum (min) value, maximum (max) value, mean, and standard deviation (std). For example, the Pregnancy (PR) attribute has a record count of 786, a mean value of 3.84, a standard deviation of 3.36, and the maximum and minimum PR values are 17 and 0, respectively. Similar statistical measurements have been computed for the remaining attributes as well. These metrics provide valuable insights into the data distribution and properties.

**TABLE 2:** Attributes information of the dataset.

Attribute	Count	Mean	Std	Min	Max
PR	768	3.84	3.36	0	17
GL		120.89	31.97	0	199
BP		69.10	19.35	0	122
ST		20.53	15.95	0	99
IN		79.79	115.24	0	846
BMI		31.99	7.88	0	67.10
DPF		0.47	0.33	0.78	2.42
AG		33.24	11.76	21	81
DB		0.34	0.47	0	1

### 3.4 Histogram of attributes

**FIGURE 2:**
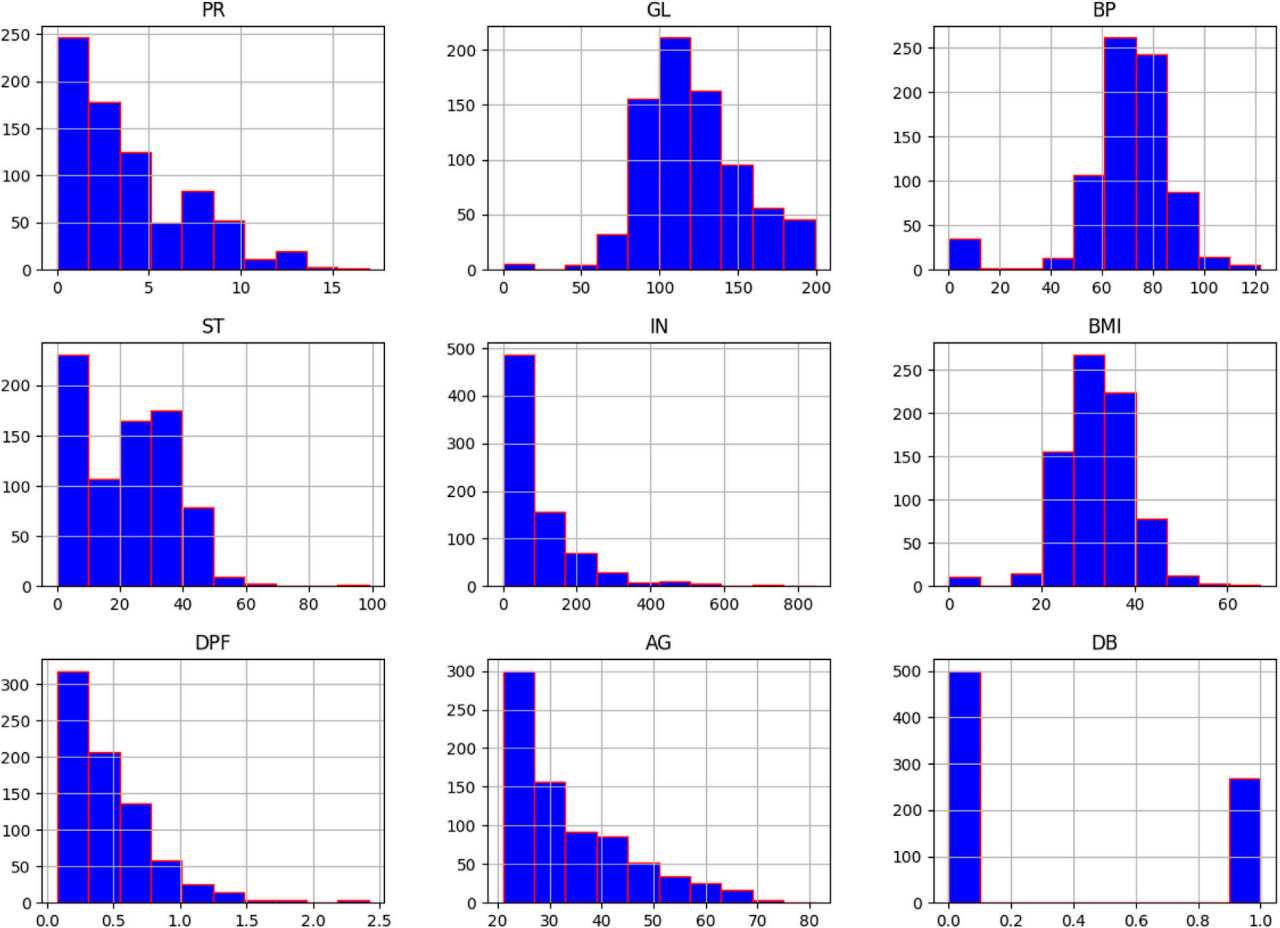
Histogram of attributes.

**FIGURE 3:**
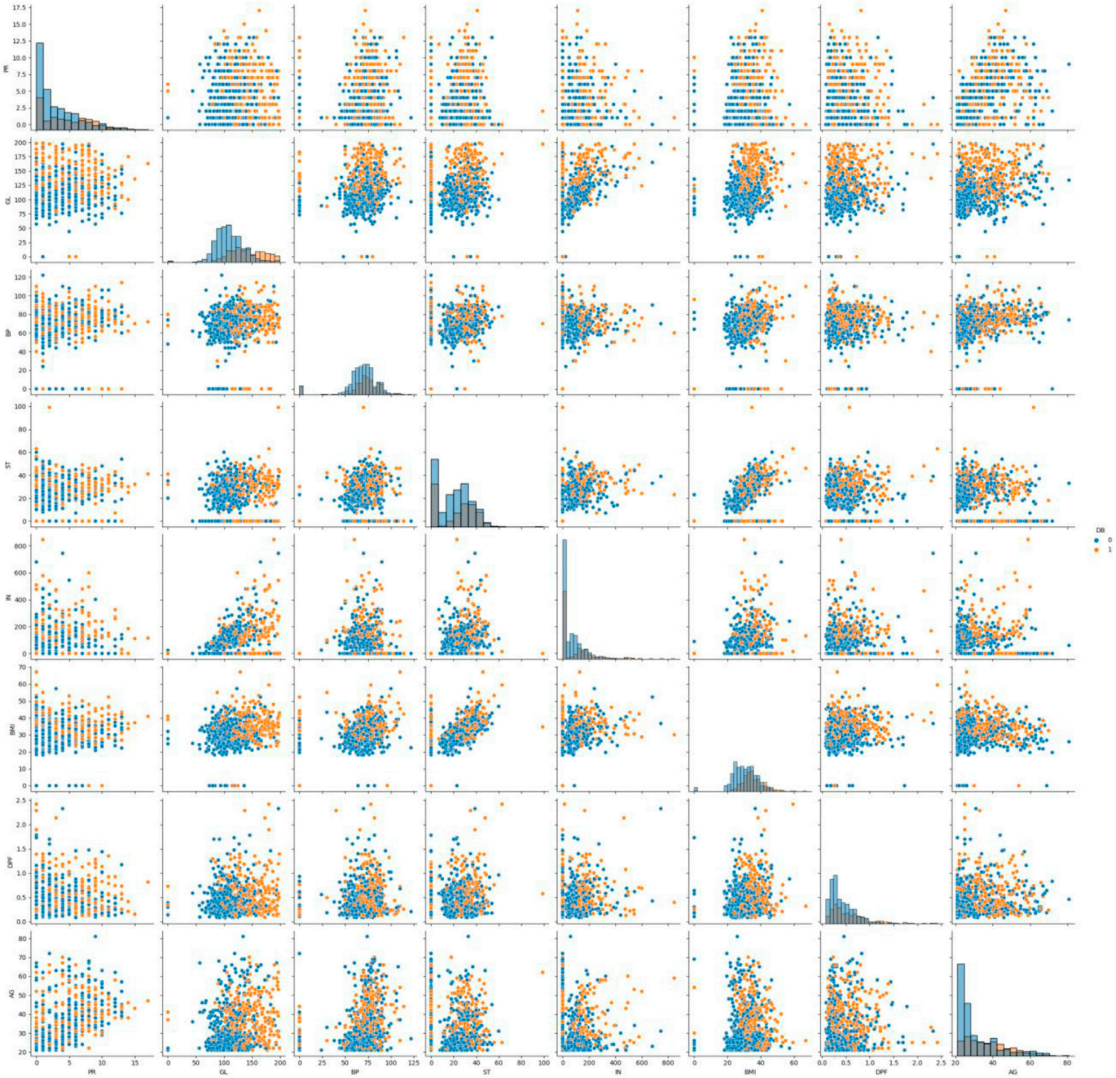
Distribution of attributes for nondiabetics and diabetics.

A histogram is a useful tool for visualising and understanding the distribution of data samples in a dataset. It provides insights into whether the data follows a uniform, normal, left-skewed, or right-skewed distribution. In Figure 2, normally distributed histograms are presented, depicting the grouping of all attributes within their respective range values. This visualisation helps to better understand the data distribution and identify any patterns or anomalies present in the dataset. The X-axis describes the input attributes, and the Y-axis presents the value of that attributes. The distribution of attributes for nondiabetics and diabetics is shown in Figure 3. In the figure, 0 (blue color) and 1 (orange color) represent nondiabetic and diabetic patients, respectively. It can be seen that in most of the attribute combinations, the tendency of being diabetic increases when their respective range values increase. For example, in the age vs. glucose level plot, we understand that patients more than 30 years with glucose levels more than 125 are more likely to be diabetic patients.

### 3.5 Boxplot for each attribute

**FIGURE 4:**
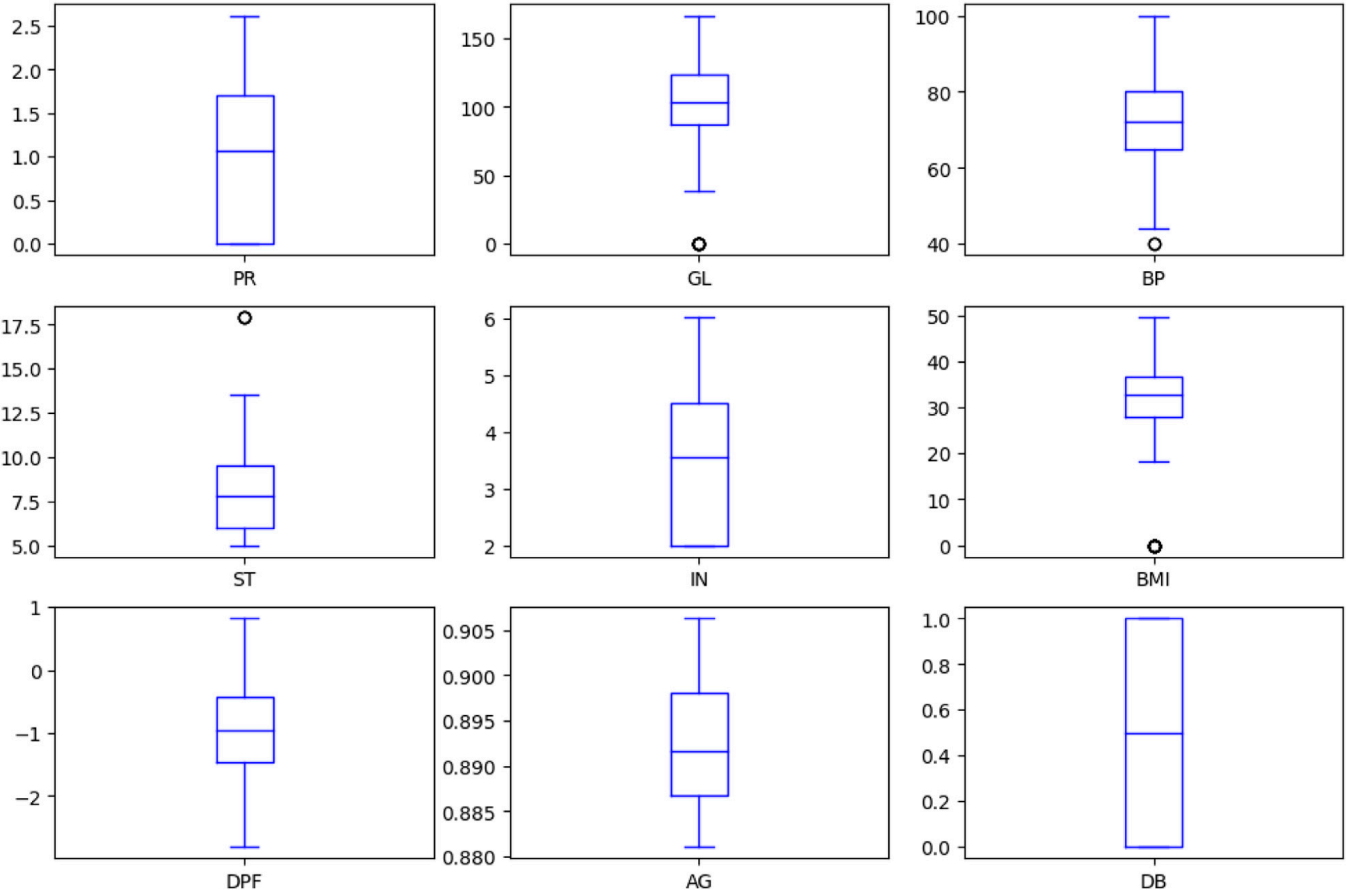
Boxplot of attributes.

Figure 4 depicts the boxplot of all the considered attributes of the dataset. It provides a good indication of how the dispersion of values is spread out. The Interquartile Range (IQR) method, based on the probability density function, has been employed to display boxplots for the characteristics to manage outliers in the dataset. This approach aids in visually representing data distribution, particularly focusing on the median, quartiles, and any potential outliers. By incorporating the IQR method, the boxplots provide valuable insights into the central tendency and variability of each attribute, while effectively addressing and visualising the presence of outliers.

### 3.6 Correlation coefficient analysis

**FIGURE 5:**
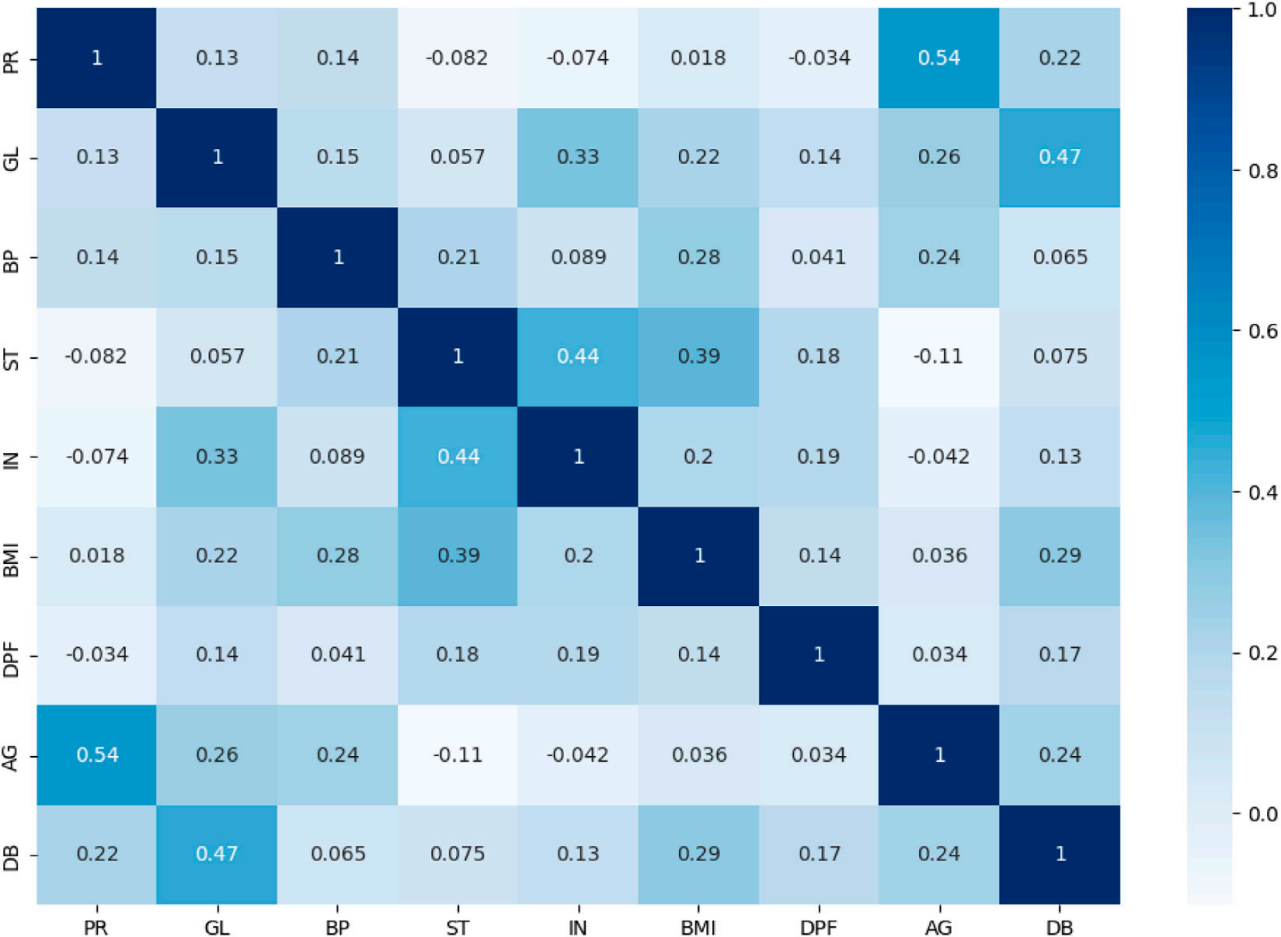
Correlation coefficient analysis.

The dataset’s attribute associations are examined and visualised using the correlation coefficient analysis (CCA) approach (Hussain and Naaz, 2021). A high correlation between the independent qualities set and the dependent attribute is desired to judge a good dataset (Ganie et al., 2023). The CCA plot of every variable used to predict disease is shown in Figure 5. The intensity and direction of the correlations between the qualities are shown by the x-axis and y-axis, which describe the range of associations and range from +1 to −1. The interdependencies between the variables in the dataset are better understood and identified thanks to this study.

## 4 Experiment, results, and discussion

The experimental minutiae and findings generated by the application of boosting algorithms for diabetes prediction are presented and discussed in this part. The outcomes obtained after using the suggested framework are methodically presented and examined. The evaluation is carried out thoroughly, considering several measures for the evaluated boosting algorithms, including accuracy, recall, precision, F1-score, micro-weighted score, average weighted score, and the receiver operating characteristic (ROC) curve. These measures give us important information about how well the boosting algorithms perform and how well they forecast diabetes.

### 4.1 System specification

The research work was conducted using an HP Z60 workstation with the following hardware specifications: Intel XEON 2.4 GHz CPU (12 core), 8 GB RAM, 1 TB hard disk, and running on Windows 10 Pro 64-bit operating system.

The tools utilised for implementation included Python as the programming language, the web-based computing platform Jupyter Notebook, and the graphical user interface-based Anaconda Navigator.

### 4.2 Data preprocessing

Data preparation is essential in creating a strong and reliable system before applying machine learning techniques to the model (Jazayeri et al., 2020). In this work, various strategies were used to manage various data preparation issues.

Firstly, missing values were located and dealt with using the data imputation method. All of the missing values were found using the isnull() function, and they were then filled using the mean and mode imputation method and the SimpleImputer() method. With this method, the mean, median, or mode of the relevant column was used to fill in the gaps left by the missing data.

To handle outliers, the IQR method was applied. The distribution of each data sample was altered using the Z-score to make the mean equal to 0. This process helped in identifying and replacing outliers in the dataset.

Furthermore, data cleaning methods were employed to address duplication, inconsistency, and corrupted data. These techniques ensured the integrity and reliability of the dataset by removing or resolving any duplicate records, inconsistent values, or corrupted data points.

By implementing these data preprocessing techniques, the dataset was prepared and optimised for subsequent machine learning methods, enhancing the quality and reliability of the analysis.

### 4.3 Data upsampling

**FIGURE 6:**
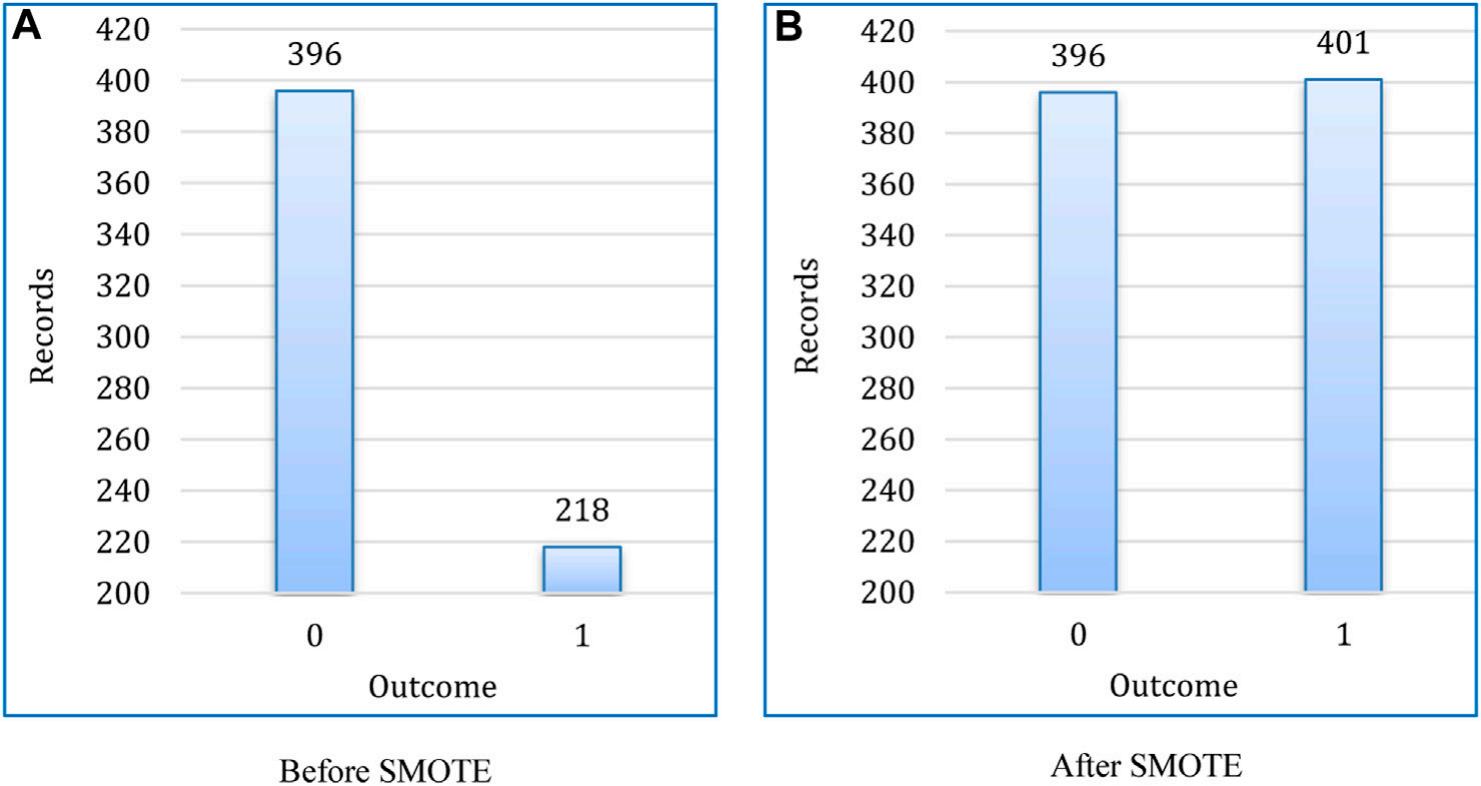
Upsampling technique for class balancing in training dataset. **(A)** Before SMOTE, **(B)** After SMOTE.

If the dataset is not balanced, machine learning and deep learning algorithms produce subpar outcomes (Ganie et al., 2023). In this work, the dataset was highly biased toward the negative class, i.e., “0-non-diabetic” over the positive class “1-diabetic.” Initially, out of 786 instances, 500 records were negative class, whereas only 268 instances were held for positive class. After splitting, we had 614 records in the training dataset, in which 396 was for non-diabetic and 218 for diabetic. To balance the training set, the SMOTE was used, as shown in Figure 6.

### 4.4 Data normalisation

Normalisation is a part of the feature scaling process that fits the data samples into a specific range. The nature of the dataset can determine the range of values. Mostly, the values fit between the range of 0–1. In our study, we used min-max scaling to bring the attribute values between 0 and 1. The mathematical expression used to perform data min-max scaling is given in Eq. 1.

$$x_{scaled}=\frac{x-x_{\min}}{x_{\max}-x_{\min}}$$ (1)

where *x* is the attribute value, and *x*_*min*_ and *x*_*max*_ denote the minimum and maximum values of *x*, respectively.

### 4.5 K-fold cross validation

K-fold cross validation is typically used to remove the biasness in the dataset. In this method, the dataset is partitioned into *k* approximately equal-sized subsets, also known as “folds”. In this experiment, applied K-fold cross validation on the training dataset and got the best result using the value of *k* as 10. The results in the following sections are based on this value.

### 4.6 Hyperparameter tuning

Hyperparameter tuning is important because it controls the training algorithm’s behavior and significantly impacts the model’s performance evaluation. Grid search and random search methods were used for hyperparameter tuning, as presented in Table 3. The listed values for each parameter for the respective algorithms were found to be the best performers in our experiment.

**TABLE 3:** Hyperparameter tuning of boosting algorithms.

Boosting algorithm	Hyperparameters
XGBoost	learning_rate = 0.01, n_estimators = 1000, max_depth = 4, min_child_weight = 8, subsample = 0.6, reg_alpha = 0.005, seed = 27
CatBoost	learning_rate = 0.010, 0.004, “depth” = 4, leaf_reg’, 1.0, min_child_samples = 1, 4, 8, 16, 32, iterations = 3000, random_state = 42
LightGBM	boosting_type = “lgbm”, class_weight = Auto, min_child_weight = 0.01, random_state = 124, num_leaves = 11, n_estimators = 1500, n_jobs = 6
AdaBoost	learning_rate = [0.0001, 0.001, 0.01, 0.1, 1.0], base_estimator = base, grid_search = GridSearchCV, param_grid = grid, parameters, cv = 5, n_jobs = n_jobs
Gradient boosting	learning_rate = 0.01, n_estimators = 100000, max_depth = 8, colsample_bytree = 0.8, reg_alpha = 0.002, scoring = roc_curve, weight = 4, subsample = 0.6, seed = 23

### 4.7 Feature importance

Based on their contribution to forecasting the output feature (target variable), the feature significance procedure assigns scores to input attributes (predicate variables) (Dutta et al., 2019). This phase is essential for machine learning or ensemble learning models to produce better predictions.

The feature significance score (F-score), which measures how frequently an attribute is used for splitting during training, is employed in this study. A characteristic, such as DPF (Diabetes Pedigree Function), with a higher F-score is considered an essential attribute since it contributes more significantly to the prediction process.

**FIGURE 7:**
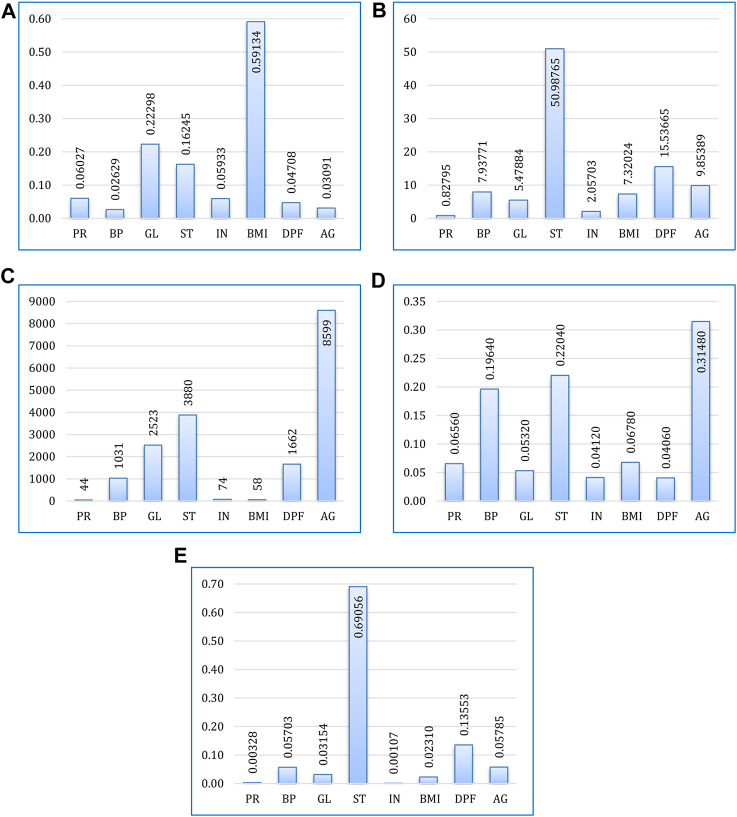
Feature importance for prediction using **(A)** XGBoost, **(B)** CatBoost, **(C)** LightGBM, **(D)** AdaBoost, and **(E)** Gradient boosting.

According to their relative F-scores for each boosting algorithm, Figure 7 displays the contribution of all attributes to the prediction task. It can be observed that overall, age, BMI, and skin thickness are the most common indicators of the patient having diabetes. Increased glucose level and high blood pressure are also a matter of concern. Out of eight features, none of them were found to be absolutely insignificant for diabetes.

### 4.8 Accuracy of classifiers

**FIGURE 8:**
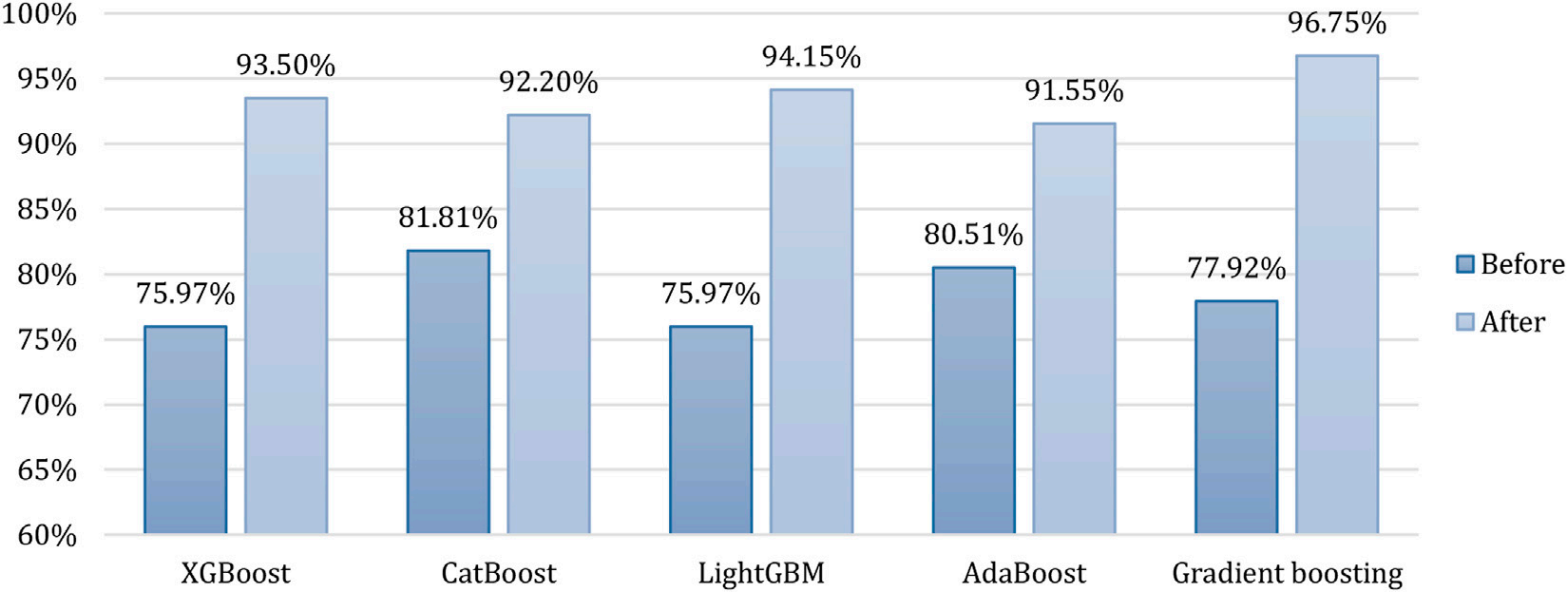
Accuracy of all the boosting algorithms before and after data processing, augmentation and hyperparameter tuning.

The testing accuracy (calculated using Eq. 2) (Pramanik et al., 2020) of the boosting algorithms (i.e., XGBoost, CatBoost, LightGBM, AdaBoost, and gradient boosting) is presented in Figure 8. The figure presents a comparison of the accuracy of the considered algorithms before and after conducting data processing, augmentation and hyperparameter tuning. It can be observed that before data processing CatBoost performed best with the highest accuracy of 81.81%. In comparison, gradient boosting emerged as the top performer after data processing, with the highest accuracy of 96.75%.

$$Accuracy=\left(TN+TP\right)/\;\left(TN+FN+TP+FP\right)$$ (2)

where TN: true negative, TP: true positive, FN: false negative, and FP: false positive.

### 4.9 Confusion matrices

**FIGURE 9:**
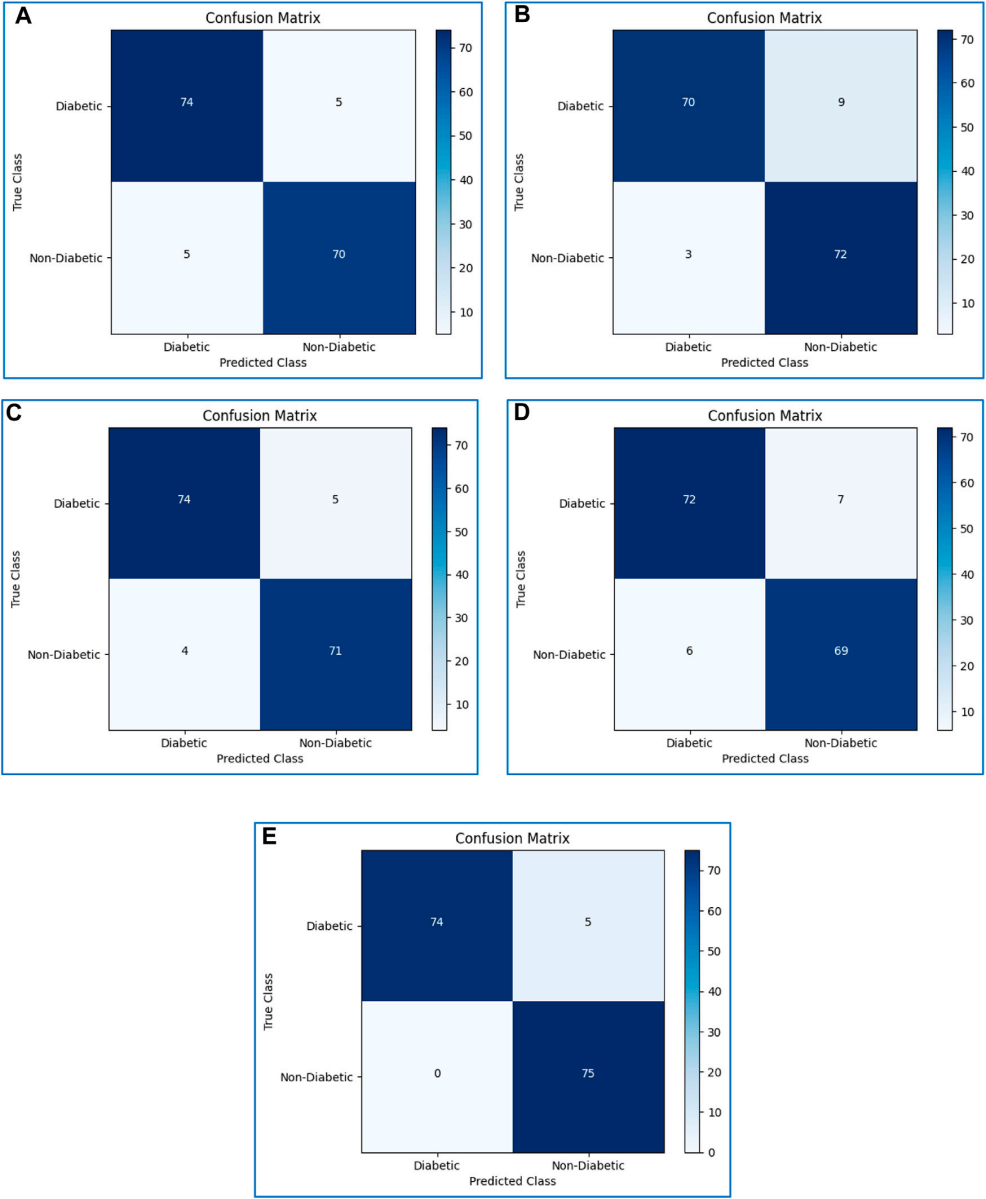
Confusion matrices of **(A)** XGBoost, **(B)** CatBoost, **(C)** LightGBM, **(D)** AdaBoost, and **(E)** Gradient boosting.

The performance evaluation of all classifiers was evaluated using a confusion matrix. The confusion matrices of all considered boosting algorithms are shown in Figure 9.

### 4.10 Other measurements

**FIGURE 10:**
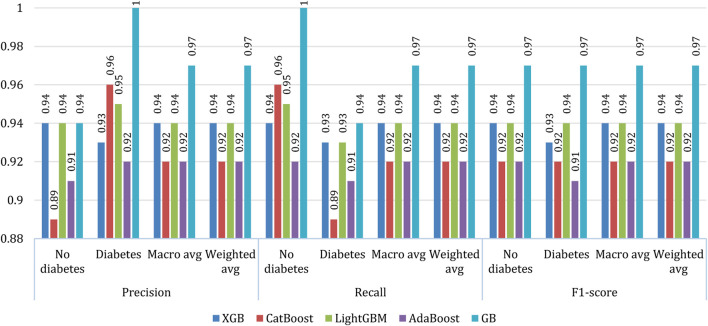
Other measurements of classifiers.

The precision (Eq. 3) (Ganie et al., 2022a), recall (Eq. 4) (Ganie and Malik, 2022b), and f1-score (Eq. 5) (Ganie et al., 2022b) of the five considered classifiers were calculated. Furthermore, the macro average and the weighted average were measured for both classes (0: no diabetes, 1: diabetes), as shown in Figure 10. On average, gradient boosting exhibited better results than other models in all respects. However, in the case no diabetes precision the performance of gradient boosting is at par with XGBoost and Light GBM. In most of the cases, XGBoost and Light GBM exhibited similar performances while in some cases, the performance of CatBoost and AdaBoost are found equivalent.

$$Precision=TP/\left(TP+FP\right)$$ (3)

$$Recall=TP/\left(TP+FN\right)$$ (4)

$$f1-score=2TP/\;\left(2TP+FP+FN\right)$$ (5)

### 4.11 ROC curve

The prediction ability of the discussed boosting algorithms is evaluated at various levels using the receiver operating characteristic (ROC) curve. On the y-axis, it displays the true-positive rate (TPR) (Eq. 7) (Pramanik et al., 2020)) and on the x-axis, the false-positive rate (FPR) (Eq. 6) (Pramanik et al., 2020). We may assess how well the models can differentiate between the two classes—0 (non-diabetic) and 1 (diabetic)—by examining the ROC curve.

$$FPR=FP/\;\left(FP+TN\right)$$ (6)

$$TPR=TP/\;\left(TP+FN\right)$$ (7)

A higher ROC curve indicates that the model performs well in differentiating between the two classes (Ganie et al., 2023). Moreover, the area under the ROC curve (AUC) is used as a measure of separability. An AUC value close to 1 indicates a good separability measure, while a value close to 0 signifies a poor measure of discrimination. A value of 0.5 suggests that the model is not effectively separating the classes.

**FIGURE 11:**
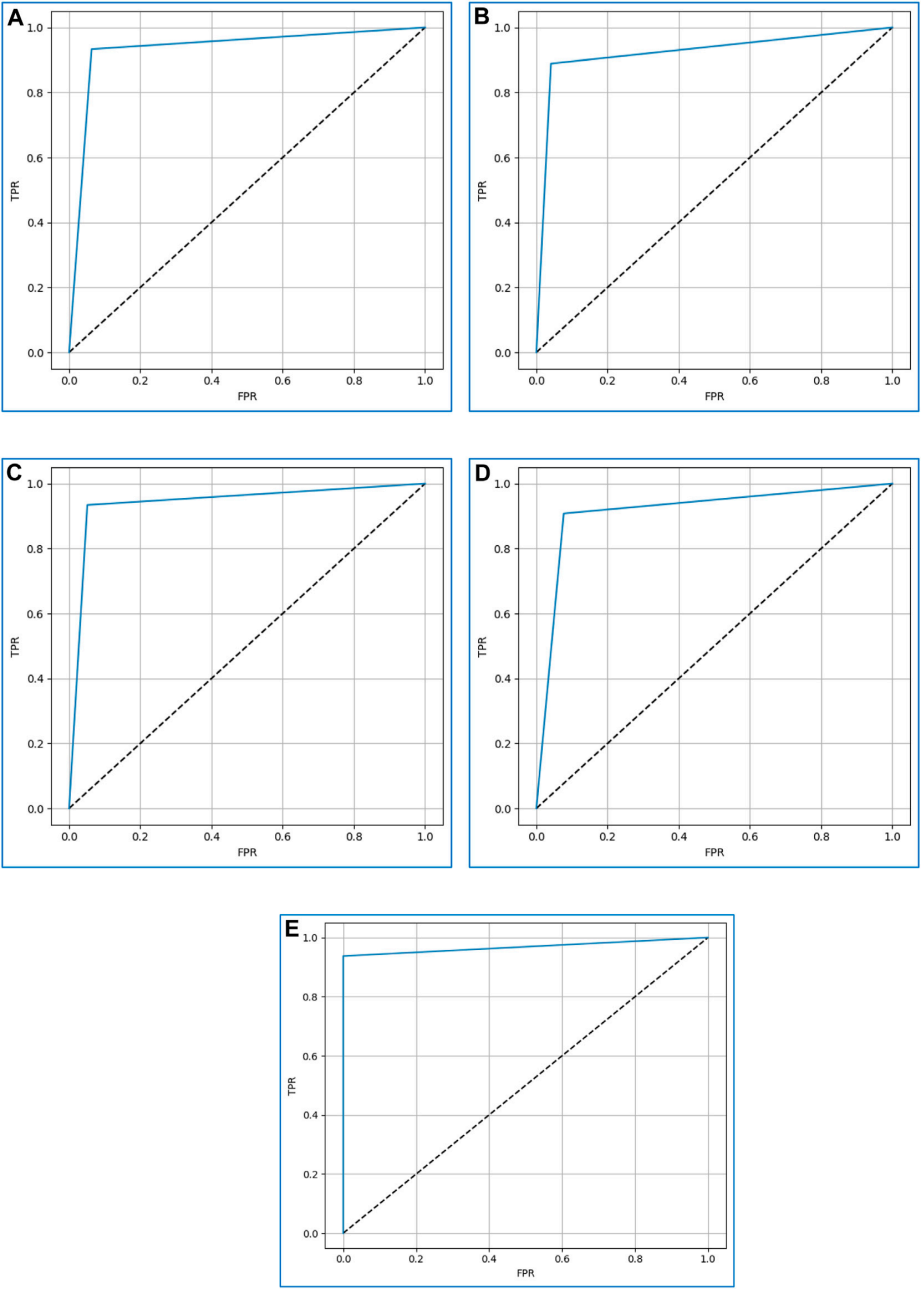
The ROC curves for **(A)** XGBoost, **(B)** CatBoost, **(C)** LightGBM, **(D)** AdaBoost, and **(E)** Gradient boosting.

Figure 11 displays the ROC curves for XGBoost, CatBoost, LightGBM, AdaBoost, and gradient boosting. Based on the curves, gradient boosting performed the best, while AdaBoost exhibited the poorest performance among the considered boosting algorithms.

### 4.12 AUPRC

**FIGURE 12:**
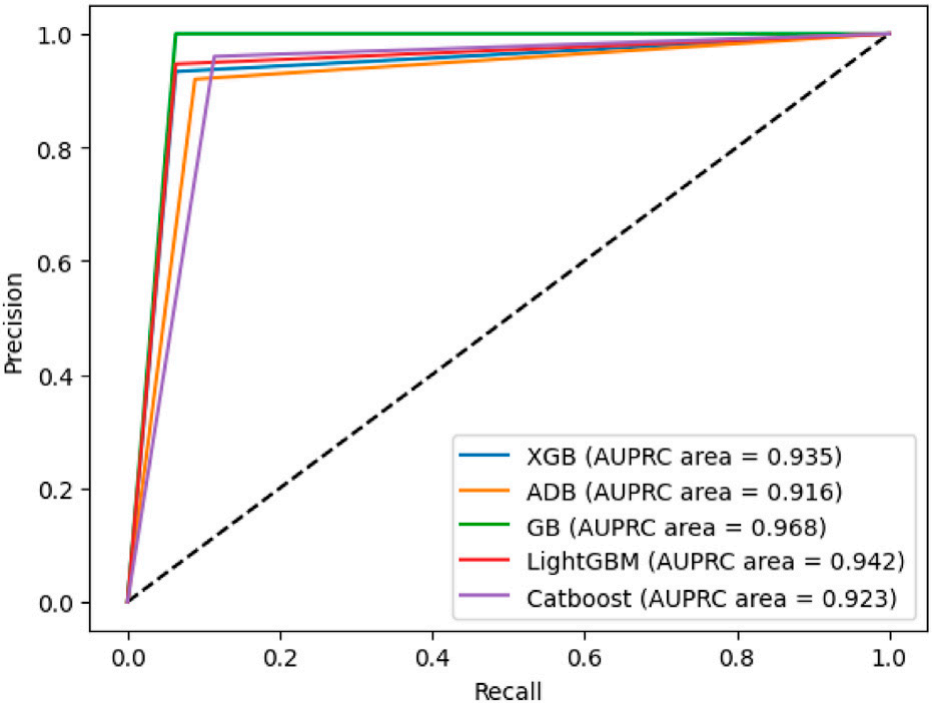
The AUPRC for the experimented boosting algorithms.

Area Under the Precision-Recall Curve (AUPRC) metric is employed to assess the machine learning model’s performance, distinguishing between a positive class and a negative class. It illustrates the relationship between precision, representing the positive predictive value, and recall, indicating sensitivity or the genuine positive rate. This curve is constructed by considering different probability thresholds for the positive class. The AUPRC for our proposed model is shown in Figure 12, from which it is observed that gradient boosting and AdaBoost have the best and worst performances, respectively.

## 5 Comparative analysis

**FIGURE 13:**
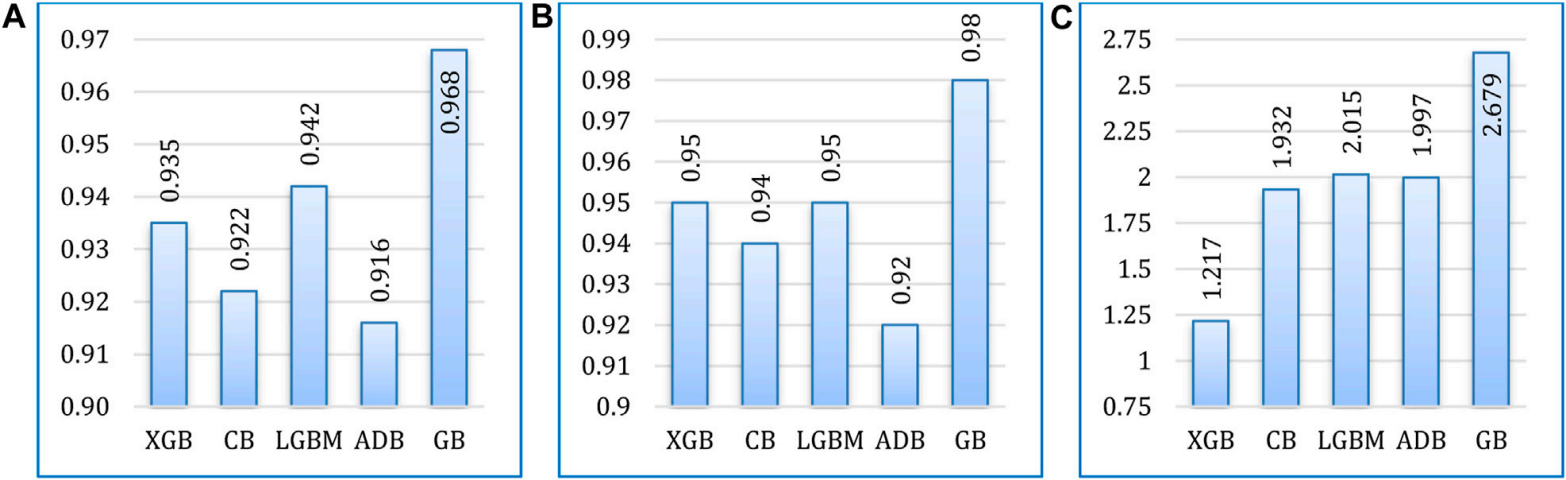
Comparative analysis of the considered algorithms in terms of **(A)** accuracy, **(B)** AUC, and **(C)** runtime (in seconds).

Figure 13 presents a comparative analysis of the five boosting algorithms considered in the experiment. The algorithms were compared in terms of accuracy, AUC value, and runtimes. Among these algorithms, gradient boosting achieved the highest accuracy rate, reaching a maximum accuracy of 96.75%. Following gradient boosting, LightGBM achieved an accuracy rate of 94.15%, AdaBoost achieved 91.55%, CatBoost achieved 92.2%, and XGBoost achieved 93.5%. In addition, gradient boosting also excels in terms of AUC. However, in terms of runtime XGBoost outperforms others, requiring the least runtime.

We compared the highest accuracy achieved by our proposed method (i.e., using gradient boosting) with several relevant literature in terms of accuracy, as shown in Table 4. The implemented processes, such as data imputation for handling missing values, detection, and box plotting for outlier elimination, could be credited with the reason for achieving improved accuracy.

**TABLE 4:** Comparison of the proposed work with existing similar works.

Research work	Adopted ensemble methods	Dataset used	Highest accuracy
Li et al. (2020)	XGBoost, XGBoost + logistic regression, data feature stitching + XGBoost	PIMA Indian diabetes dataset	80.20% with data feature stitching + XGBoost
Mahabub (2019)	kNN, AdaBoost, decision tree, random forest, support vector classification, gradient boosting, multilayer perceptron, XGBoost, gaussian naive Bayes	Do	84.42% with multilayer perceptron
Mushtaq et al. (2022)	kNN, random forest, naive Bayes, SVM, gradient boosting, logistic regression, and voting classifier	Do	81.30% with voting classifier
Beschi Raja et al. (2019)	Neural networks, random forest, and GBC	Do	76.10% with GBC
Khan et al. (2021)	Gradient boosting, hybrid K-mean, J48, decision tree, deep learning, naive Bayes, and ANN	Do	92% with gradient boosting algorithm
Singh et al. (2021)	XGBoost, random forest, SVM, neural network, and decision tree	Do	95% with XGBoost and random forest
Hasan et al. (2020)	kNN, decision trees, random forest, AdaBoost, naive Bayes, XGBoost, and multilayer perceptron	Do	88.84 with AdaBoost + XGboost
This paper	XGBoost, CatBoost, LightGBM, AdaBoost, and gradient boosting	Do	96.75% with gradient boosting

## 6 Conclusion and future scope

In this research, we investigated the effectiveness of five boosting algorithms, namely, XGBoost, CatBoost, LightGBM, AdaBoost, and gradient boosting, for predicting diabetes disease. Various preprocessing techniques, such as imputation, Z-score, and cleaning methods, were applied to improve the quality of the dataset. Additionally, to enhance disease prediction, data normalisation, upsampling, and hyperparameter tuning were performed.

According to the experimental findings, gradient boosting had the greatest accuracy rate of 96%. Additionally, it did well in terms of other evaluation criteria like ROC curve, precision, recall, and f1-score. The feature importance technique revealed how independent features contributed to the outcome of the final prediction.

Furthermore, when compared to similar related efforts, the suggested framework performed better than existing systems. Other ensemble learning strategies, such as bagging and stacking, can be added to further increase the quality of the outcomes. To increase the scope of this research, the proposed method can also be used for other healthcare datasets with comparable features.

In future studies, exploring deep learning techniques could lead to better detection and prediction of diabetes. These advancements in machine learning and deep learning can contribute to more accurate and efficient healthcare solutions.

## Data Availability

The original contributions presented in the study are included in the article/Supplementary Material, further inquiries can be directed to the corresponding authors.
